# Medical school faculty discontent: prevalence and predictors of intent to leave academic careers

**DOI:** 10.1186/1472-6920-7-37

**Published:** 2007-10-14

**Authors:** Steven R Lowenstein, Genaro Fernandez, Lori A Crane

**Affiliations:** 1 Division of Emergency Medicine, Box B-215, University of Colorado School of Medicine, 4200 East Ninth Avenue, Denver, Colorado 80262, USA; 2 Department of Preventive Medicine and Biometrics, University of Colorado School of Medicine, 4200 East Ninth Avenue, Denver, Colorado 80262, USA; 3 Office of the Dean, University of Colorado School of Medicine, 4200 East Ninth Avenue, Denver, Colorado 80262, USA

## Abstract

**Background:**

Medical school faculty are less enthusiastic about their academic careers than ever before. In this study, we measured the prevalence and determinants of intent to leave academic medicine.

**Methods:**

A 75-question survey was administered to faculty at a School of Medicine. Questions addressed quality of life, faculty responsibilities, support for teaching, clinical work and scholarship, mentoring and participation in governance.

**Results:**

Of 1,408 eligible faculty members, 532 (38%) participated. Among respondents, 224 (40%; CI95: 0.35, 0.44) reported that their careers were not progressing satisfactorily; 236 (42%; CI95: 0.38, 0.46) were "seriously considering leaving academic medicine in the next five years." Members of clinical departments (OR = 1.71; CI95: 1.01, 2.91) were more likely to consider leaving; members of inter-disciplinary centers were less likely (OR = 0.68; CI95: 0.47, 0.98). The predictors of "serious intent to leave" included: Difficulties balancing work and family (OR = 3.52; CI95: 2.34, 5.30); inability to comment on performance of institutional leaders (OR = 3.08; CI95: 2.07, 4.72); absence of faculty development programs (OR = 3.03; CI95: 2.00, 4.60); lack of recognition of clinical work (OR = 2.73; CI95: 1.60, 4.68) and teaching (OR = 2.47; CI95: 1.59, 3.83) in promotion evaluations; absence of "academic community" (OR = 2.67; CI95: 1.86, 3.83); and failure of chairs to evaluate academic progress regularly (OR = 2.60; CI95: 1.80, 3.74).

**Conclusion:**

Faculty are a medical school's key resource, but 42 percent are seriously considering leaving. Medical schools should refocus faculty retention efforts on professional development programs, regular performance feedback, balancing career and family, tangible recognition of teaching and clinical service and meaningful faculty participation in institutional governance.

## Background

*For the first time in recent history, academic medical centers need to be concerned about attracting the next generation of faculty and leaders as well as losing productive faculty who are disenchanted and struggling in the current academic setting" *[1].

Arnold Rice Rich, who served as professor of pathology at the Johns Hopkins University School of Medicine from 1919–1958, observed that, in his day, medical school faculty could enjoy "the element of repose, the quite pursuit of knowledge, the friendship of books, the pleasures of conversations and the advantages of solitude." [2] There is a general consensus among medical school faculty that those times are gone. Today, faculty face relentless pressure to generate revenues from direct patient care or grants [3-5]. Administrative and regulatory requirements are ever-expanding. And, as academic medical centers increasingly emphasize payer mix, managed care, business models and the "bottom line," it is becoming more difficult to find time to teach, balance family and career, keep up with advances in medicine and science, gather with colleagues and engage in meaningful scholarship." [2-13] Few faculty members are able to enjoy the quiet repose that Professor Rich described. Rather, studies reveal widespread disillusionment and a high rate of job turnover among medical school faculty members [14]. Among graduating medical students and residents, there is a declining interest in academic medical careers [15,16].

We conducted this survey of medical school faculty in order to learn more about the sources of faculty discontent. The survey was designed with two specific objectives: First, we sought to measure the "prevalence of discontent" by asking faculty about their career progress and their intent to leave academic medicine. Second, we sought to identify key "predictors of discontent" from a variety of domains, including faculty characteristics, quality of life, resources for faculty development, institutional support for teaching, research and clinical practice, and participation in institutional governance. Valid and representative data about faculty attitudes and experiences, and about intent to leave academic medicine, may prove useful in sharpening the focus of faculty development and support programs.

## Methods

This survey was conducted in September, 2001 at the University of Colorado School of Medicine. The target population included all full-time faculty at the rank of instructor or above. Part-time (less than 0.5 full-time equivalent), volunteer and adjunct faculty were excluded.

### Setting

The University of Colorado School of Medicine (SOM) is public institution engaged in the traditional missions of teaching, research and clinical and community service. At the time of the survey there were 1,408 members of the full-time faculty, employed by the University or by one of three affiliated teaching hospitals.

The SOM's promotion and tenure policies were reformed in 1997, in order to recognize the growing numbers of clinician-educators [5] First, the SOM created a single, tenure-eligible promotion track for all faculty, including educators, clinicians and basic scientists; promotion and tenure awards were made separate processes. Second, citing Ernest Boyer and the Carnegie Foundation's 1990 Report, *Scholarship Reconsidered: Priorities of the Professoriate *[17], the SOM expanded the definition of scholarship to include the discovery, integration, teaching and application of knowledge. A comprehensive promotion matrix was developed, which included more than sixty examples of alternative forms of scholarship that are pertinent to clinician-educators; the matrices made it clear that clinical case reports, book chapters, review articles, case simulations, evidence-based patient care guidelines and innovative teaching syllabi are appropriate types of scholarship for academic physicians who are fully engaged in the practice or teaching of clinical medicine. Third, teaching and clinical service were given greater weight and parity in the promotion process, and faculty were encouraged to submit evidence of their accomplishments using clinical, teaching and service portfolios. Fourth, the School clarified that extensions to the 7-year promotion time-clock would be granted by the Dean for various career interruptions, illnesses, family obligations or periods of part-time service. Beginning in 2000, several faculty development initiatives were also implemented, including an annual New Faculty Career Building Workshop, regular "Promotion 101"courses, an expanded Faculty Affairs web site and publication of the School of Medicine *Faculty Success Newsletter*.

### Survey Instrument

The 75 survey questions were written by a committee of faculty members and administrators. After pilot testing, the questions were revised to improve clarity and face validity. The survey was web-based and anonymous, and it was completed over a three-week period. The survey was conducted as part of the School of Medicine's administrative preparation for an accreditation visit by the Liaison Committee on Medical Education. Although the survey was not reviewed or approved by the Institutional Review Board, faculty were notified that participation was entirely voluntary and that only school-wide aggregate data would be presented.

The survey included demographic variables addressing gender, minority status, academic rank, employment status, highest degree, departmental affiliation and faculty role. Departments were classified as basic science or clinical. To determine "faculty role," respondents categorized their primary duties as clinician-educator, clinician-researcher or primary researcher.

The remaining questions were designed to measure faculty experiences and attitudes in six different domains: Quality of life (including work-family balance); faculty development (for example, mentoring programs and performance feedback); participation in institutional governance; and adequacy of support and resources for scholarship, teaching and clinical practice. For each item, faculty members were asked whether they agreed or disagreed with a statement (for example, *"In my job I have enough time to teach"*). Each statement was followed by Likert scale response options ranging from 1 ("strongly agree") to 5 ("strongly disagree"). For ease of presentation, ordinal responses were collapsed into dichotomous categories: "Agree" (including "strongly agree" and "agree") or "disagree" ("don't know," "disagree," or "strongly disagree").

### Outcome Variables

The principal objective of this study was to measure the proportion of SOM faculty who were seriously considering leaving academic medicine and to identify the factors that influenced that consideration. The survey included the following statement: *"I am seriously considering leaving academic medicine in the next five years." *Those who "strongly agreed" or "agreed" were classified as "seriously intending to leave." Those who answered "don't know," "disagree" or "strongly disagree" were classified as "no intent to leave."

A second objective was to measure career progress. Respondents who "agreed" or "strongly agreed" with the statement, *"My academic career has been progressing at a satisfactory rate since I joined the School of Medicine" *were classified as "making satisfactory career progress."

### Data Analysis

The analysis proceeded in two steps. First, survey responses were summarized using proportions and 95 percent confidence limits. Second, bivariate analyses were performed to test for associations between survey responses and the principal outcome variable, "serious intent to leave academic medicine." To measure the strength of the associations, crude odds ratios and 95% confidence limits were calculated. Survey participants were not required to answer every question, and question-specific response rates ranged from 88 to 100 percent. The high frequency of missing responses for specific questions precluded generation of multivariate models.

## Results

### Characteristics of Survey Participants

Of 1,408 eligible faculty members, 532 (38%) participated. The majority of respondents were men (60%) and non-Hispanic whites (90%). Most held M.D. degrees (68%) and were members of clinical departments (84%). The distribution according to faculty rank was relatively balanced: Instructors (14%); assistant professors (32%); associate professors (27%); and full professors (27%). With respect to faculty roles, 45 percent of respondents said they were clinician-researchers; smaller proportions were primary researchers (31 percent) or clinician-educators (24 percent). Over one-third (37 percent) reported they were members of an inter-disciplinary center in addition to their home department.

There were some differences between the survey participants and the faculty-at-large. Fifty-four percent of respondents were associate or full professors, compared with 43 percent among all eligible faculty. Females were slightly over-represented among survey respondents (40% vs. 36%). Participants and eligible faculty were similar with respect to department type.

### Survey Reliability

The internal consistency of the questions in each domain was examined using the Cronbach's alpha coefficient. The coefficients indicated a moderate or high level of consistency among the responses: Quality of life (0.77); faculty development (0.69); participation in institutional governance (0.81); and institutional support for scholarship (0.50), teaching (0.65) and clinical excellence (0.76).

### Intent to Leave Academic Medicine and Career Dissatisfaction

Among the 561 survey respondents, 224 (42%; CI95: 0.38, 0.46) reported they were "seriously considering leaving academic medicine in the next five years." A similar proportion (40%; CI95: 0.35, 0.44) reported that their career was not progressing satisfactorily.

### Predictors of Serious Intent to Leave Academic Medicine

Members of clinical departments (OR = 1.71; CI95: 1.01, 2.91) were more likely to consider leaving academic medicine; members of interdepartmental centers were less likely to consider leaving (OR = 0.68; CI95: 0.47, 0.98). Gender was not a significant predictor of intent to leave (OR for women = 1.39; CI95: 0.96, 1.99). Intent to leave academic medicine was not associated with minority status, faculty role (clinician, clinician-investigator or primary investigator) or highest degree. In addition to these demographic variables, the "top ten" experiential predictors of serious intent to leave academic medicine are listed in table 1.

**Table 1:** Top ten predictors of "serious intent" to leave academic medicine

	**Prevalence (%)**	OR (95% CI)*
**Quality of Life**		

It is not easy to balance family responsibilities with career development	66	3.52 (2.34,5.30)
My department lacks a sense of academic community	43	2.67 (1.86, 3.83)

**Teaching**		

The School does not adequately recognize innovative and high quality teaching	75	2.47 (1.59, 3.83)
My department does not foster and reward teaching excellence	63	2.40 (1.64, 3.51)

**Clinical Service**		

Time for clinical services is not recognized in evaluation and promotion criteria	83	2.73 (1.60, 4.68)
My department does not support excellence in clinical service	58	2.54 (1.74, 3.69)

**Faculty Development**		

My department lacks an effective program of faculty development	69	3.03 (2.00, 4.60)
My department has not evaluated my academic progress regularly	41	2.60 (1.80, 3.74)

**Governance**		

The Dean and Chancellor do not understand demands placed on faculty and support them	56	2.84 (1.75, 4.59)
Faculty do not have the opportunity to comment on the performance of institutional leaders	88	3.08 (2.07, 4.72)

*Odds Ratio expresses the increased likelihood of "Serious intent to leave academic medicine" when faculty member "Agrees" or "Strongly Agrees" with this statement.

## Discussion

The academic and financial success of a medical school depends upon the faculty – how well they teach, the quality of their clinical care and service, and their contributions to scholarship and discovery. However, several recent studies have indicated that medical school faculty are disillusioned and, possibly, endangered. Career satisfaction and success, especially for clinician-educators, are threatened by the lack of time for teaching, scholarship and personal and professional self-renewal [1-7,10,13,18-21]. Faculty success may also be threatened by fundamental clashes between faculty and institutional values [1,3,6]. Carr et al observed that "faculty often feel powerless against the behemoth of a large institution and often have the perception that the large, bureaucratic nature of an institution is unresponsive to individuals, unwilling to negotiate and only 'looking for a bargain,' especially in times of constrained resources." [1] For medical school faculty, wrote Strasburger, "These are the times than can try one's soul." [22]

Whatever the root causes of faculty discontent, the consequences are real. Medical students and residents are influenced in their career choices by the attitudes of their faculty preceptors; [16,18-25] today, the percentage of medical school and residency graduates seeking academic medical careers is declining [1,15,16,26]. Medical school faculty turnover is high, now averaging 8–10 percent per year; attrition is especially high among women and minority faculty members and among clinicians [1,14,27-29]. In one study of fellowship-trained primary care physicians who had left their academic positions, 71 percent said it was "unlikely" or "very unlikely" that they would ever return to academic medicine. [30] The costs of faculty turnover and replacement are a significant burden to medical schools, averaging $110,000 to more than $900,000 per faculty replacement, according to various estimates [1,15,28,31]. In the clinical arena, job satisfaction is positively correlated with patient satisfaction and quality of care [24,32,33]. Obviously, medical schools must pay attention to the sources of faculty discontent.

The current study was designed to provide a snapshot of faculty discontent at one medical school. Forty-two percent of all faculty who responded to this confidential survey reported they are seriously considering leaving academic medicine in the next five years. In addition, forty percent of faculty respondents reported that their career was not progressing satisfactorily. A similar, disquieting result was reported by Fried et al, following a survey of faculty at the Johns Hopkins School of Medicine [34].

The predictors of intent to leave academic medicine included a wide spectrum of career impediments, affecting men and women, and clinicians and basic scientists, alike. In this study the "top ten" experiential predictors of serious intent to leave fell into 5 general categories. In some respects, these results are similar to earlier studies.

### Faculty Development Programs

In the current study, lack of constructive and timely feedback from department heads was associated with intent to leave academic medicine. Forty-one percent of respondents stated that they did not receive regular, effective feedback and that chairs or other senior faculty "did not evaluate [their] academic progress regularly." Previous studies have indicated that trust, open communication and regular feedback with division or department heads are strongly associated with career satisfaction [28,31].

In the current study, absence of mentoring was not a "top ten" predictor of intent to leave academic medicine. Nonetheless, absence of mentoring was prevalent (55% of respondents) and was strongly associated with intent to leave (OR = 1.76; CI95: 1.23, 2.51). Several studies have emphasized the vital importance of mentoring, especially for junior faculty, women and minorities [1,3,8,15,35]. Strong mentoring relationships are positively associated with career satisfaction and progress, research productivity and "successful acculturation in academic medicine." [1,3,8] In one study, faculty who participated in a collaborative, peer-based mentoring program were more likely to decide to remain in academic medicine [8].

Faculty development programs, which emphasize mentoring, career planning, performance feedback, establishing colleague networks and connectedness and acculturation to one's school and university, are effective interventions that improve faculty satisfaction, productivity, institutional loyalty and retention [1,3,14,15]. Even when fully funded to serve hundreds of faculty members, they are also less expensive than the costs of faculty turnover [8,31]. Unfortunately, faculty development offices and programs are under-developed or under-utilized in many medical schools [15,31,36].

### Support for clinician-educators

Serious intent to leave academic medicine was strongly associated with the belief that high quality teaching and exemplary clinical service were not rewarded by departments or during promotion and tenure reviews by the School of Medicine. In addition, intent to leave was associated with the perception that the institution did not provide adequate services and facilities to enable faculty to provide exemplary clinical care to patients. Although clinician-educators are vital to the success of modern medical schools, their faculty roles are often overlooked. Several studies have compared the careers of clinician-educators with those of basic and clinical scientists. Clinician-educator career pathways are routinely characterized by less frequent feedback from department chairs, poorer understanding of institutional promotion criteria, inadequate mentoring, less protected time for scholarship than promised, lower rates of promotion and lower levels of overall career satisfaction [4,21,37,38].

### Balancing career and family

Problems balancing family and career responsibilities emerged as a prevalent problem and was the strongest predictor of faculty discontent of any variable we studied. This is consistent with existing literature [1,9,15,30,39]. Carr et al recently pointed out that "the chronic conflicts between jobs and families in academic medicine are increasingly surfacing as [men and women] ... become less willing sacrifice balance for the sake of an academic career." [1] Today's junior faculty will work hard to meet the demands of the laboratory, classroom, clinic and health care system; but increasingly, they also seek a fuller life outside of medicine [15].

In the current study, gender was not a significant predictor of intent to leave academic medicine. Nor was status as a clinician-educator. The relatively high level of career satisfaction among women may reflect the promotion and tenure policies of the SOM, which do not penalize faculty members for career interruptions or part-time service. Clinician-educators may also enjoy a higher level of satisfaction (compared with career satisfaction at other medical schools) because they are recognized and rewarded for their commitment to clinical service and teaching. The broad definition of scholarship and the principles articulated by Boyer in *Scholarship Reconsidered *[17] have played a significant role in shaping faculty roles and rewards; among all clinician-educators who seek promotion to Associate Professor, the success rate is 96 percent [5].

### Networks of Colleagues

Our study also highlights the importance of colleague relationships. Forty-three percent of faculty said their departments did not foster a "strong academic community." A related variable, "lack of satisfying colleague relationships" was also prevalent and a strong predictor of intent to leave (OR = 2.16; CI95 = 1.46, 3.07). In a recent study of faculty vitality at the University of Minnesota Medical School, only 52 percent of faculty felt they had a network of supportive colleagues in their own department. Less than half of faculty reported having even one "weekly substantive teaching or research conversation" with any colleague – in their own department, in the medical school or anywhere in the University system [3]. The connection between colleague relationships and career satisfaction should not surprise anyone. Studies have demonstrated repeatedly that having a network of productive colleagues is one of the strongest predictors of research productivity, publications, career satisfaction, advancement and retention in academic faculty positions [3,9,35,40,41]. In one survey of academic physician-educators, the primary source of career vitality was "their association with people," including learners, mentors and colleagues [9]. Carr et al observed that "The greatest danger for junior faculty in academic medicine is isolation." [1] Importantly, faculty in our study were less likely to consider leaving academic medicine if they were affiliated with an inter-departmental research or clinical center; perhaps, this is a reflection of closer colleague networks and a stronger sense of academic community.

### Participation in institutional governance

The current study highlights an additional predictor of faculty discontent – lack of open communication with institutional leaders and an effective voice in governance. More than 55% of respondents reported they did not have adequate opportunities to participate in school governance. Only 12 percent of faculty members felt they could comment on the performance of institutional leaders, and just 9 percent felt they could influence the allocation of resources. These beliefs were strong predictors of intent to leave academic medicine. According to one faculty member in our survey, "There are opportunities [in our school] for faculty to voice opinions, but it isn't clear that anybody at the top is listening." Others have also suggested that medical faculty deserve a stronger role in governance, at least in part because a large fraction of medical school's budget derives from their earnings [42,43]. Faculty who feel involved in school governance may be more likely to "stay connected" to their institution. Political clashes and disagreements with leaders are a major reason faculty leave [30].

### Limitations

We acknowledge several important limitations of this study. First, the survey was conducted at a single public, research-intensive medical school in a Western state. Most participants were non-Hispanic whites, and the vast majority worked in clinical departments of the School. Our results may not be generalizable to other medical schools that differ in size, mission or geographic locale. A second major limitation is the response rate of 38 percent. Although participants and non-participants were similar in most demographic characteristics, non-response bias cannot be excluded. Third, the survey does not allow analysis of subsets of faculty members according to race, ethnicity or gender. These are important variables, as women and minority faculty members typically have fewer mentors, role models, colleague relationships and other resources that positively influence career success. In prior studies, minority faculty have also reported lower career satisfaction, greater isolation from mentors, colleagues and institutional leaders and a slower pace of academic progress, even after controlling for academic productivity and seniority [1,35,44-47]. Minority faculty also are more likely to leave academic medicine [1]. Finally, there are important limitations that derive from the nature of the survey. All of the data were based on self-report, and none of the questions was tested rigorously for criterion, content or construct validity. However, internal reliability analysis indicated that the questions adequately reflected specific domains. Finally, the outcome variable "serious intent to leave academic medicine" was not verified using faculty departure statistics.

## Conclusion

Medical schools must pay attention to the sources of faculty discontent. Bland has warned that "institutions can no longer take a laissez faire attitude [toward] faculty and institutional vitality, if they hope to retain faculty who are creative and successful in their work." [3] As noted earlier, programs aimed at ensuring faculty vitality, productivity and institutional loyalty are likely to be far more cost-effective than continual recruitment and retraining [1,8,14,31].

These data prompt several recommendations. First, medical school faculty and administrators should develop and conduct their own faculty needs assessments. Conducting a review of the "top ten" predictors of faculty discontent and intent to leave can assist medical schools to measure faculty vitality and sharpen the focus of their faculty development programs. Schools should regularly monitor turnover and attrition rates and compare these with national benchmarks. Periodic surveys can be conducted confidentially, rapidly and inexpensively using web-based technologies.

Second, the demographic trends are obvious. The junior faculty pipeline is increasingly filled with members of Generation X, men and women who define career success holistically and frequently take a longer view of their career trajectories [15]. They are likely to benefit by attention paid to career and family balance, with more flexible schedules, more opportunities to work part-time, and relaxation of promotion or tenure clocks [1,15,39,48,49].

Stronger mentoring and performance feedback by department chairs, and stronger networks of colleagues, can enhance faculty career satisfaction and retention. Chairs should be held accountable for holding regular meetings with faculty and providing constructive feedback and guidance regarding academic progress. Faculty also need release time to attend skill- and career-building workshops and seminars. According to Bland et al, "individuals' [academic] success depends on their knowledge, skills and motivation, but also hinges on the depth and breadth of support provided by their home institutions." [50] That support typically includes effective mentoring programs, protected work time, substantive communication among colleagues and "brokered" faculty development opportunities, all of which are found routinely in successful research-intensive enterprises [50].

Schools that have not already done so should consider revisions of academic promotion guidelines so that they recognize and reward faculty members for the jobs they are asked to do [5,7,13,18,20,34]. In particular, faculty express a strong desire that high quality teaching and clinical service be recognized and rewarded, and not marginalized or subordinated to other traditional yardsticks, such as grant acquisition, peer-review publications or evidence of a national reputation [5,7,13,18]. In our survey, one faculty member observed that, for clinician-educators, "excellent patient care and teaching should be more than enough to promote; it's our job."

Finally, there must be a strong emphasis on shared governance. This survey indicates that faculty members and the institution may benefit if faculty are provided with more tangible opportunities to participate in school-wide governance; such opportunities should include the means to influence decision-making, resource allocation and selection and evaluation of school leaders.

In a general sense, these results suggest the importance of an array of institutional features and faculty supports: Chairs who provide constructive feedback; institutional and departmental commitments to mentoring and career development; opportunities to interact with successful and influential colleagues; support and recognition for teaching and clinical service; and effective participation in institutional governance, including shared decision-making, evaluation of leaders, resource allocation and program planning. These steps will not only help empower individual faculty and aid them in their careers, but will also prove good for the institution, as they boost faculty loyalty and the institution's resiliency and leadership capacity [1,15].

## Abbreviations

SOM, School of Medicine

## Competing interests

The author(s) declare that they have no competing interests.

## Authors' contributions

SRL conceived of the study. All authors participated equally in the design of the study and in the interpretation of the data. GF and LAC performed the statistical analyses. SRL and GF prepared and edited the manuscript. All authors read and approved the final manuscript.

## Pre-publication history

The pre-publication history for this paper can be accessed here:


